# Use of a Polyethylene Bag to Reduce Perioperative Regional and Whole-Body Heat Losses in Low-Birth-Weight Neonates

**DOI:** 10.1155/2017/8243184

**Published:** 2017-07-25

**Authors:** Pierre Tourneux, Estelle Durand, Amandine Pelletier, Laurent Ghyselen, Véronique Bach, Jean-Pierre Libert

**Affiliations:** PériTox, UMR-I 01, Centre Universitaire de Recherche (CURS), Université de Picardie Jules Verne (UPJV), Site CHU, Avenue Laënnec, Salouel, 80000 Amiens, France

## Abstract

In the delivery room, wrapping a low-birth-weight neonate (defined as ≤2.499 g) in a polyethylene bag reduces the risk of hypothermia. However, extended use of the bag (e.g., during neonatal surgery) might conceivably increase the risk of thermal stress and thus body overheating. Here, we assessed the efficacy of a polyethylene bag in infants assigned to wrap (W) or nonwrap (NW, control) groups during placement of a percutaneous vena cava catheter by applying a new mathematical model that calculates heat exchanges for covered and uncovered body segments. At the end of the placement procedure, the W and NW groups did not differ significantly in terms of whole-body heat loss (15.80 versus 14.97 kJ·h^−1^·kg^−1^, resp.), whereas the abdominal skin temperature was slightly but significantly higher (by 0.32°C) in the W group. Greater evaporation in the W group (2.49 kJ·h^−1^·kg^−1^) was primarily balanced by greater whole-body radiant heat loss (3.44 kJ·h^−1^·kg^−1^). Wrapping the neonate in a polyethylene bag provides a small thermal benefit when catheter placement takes a long time. Given that polyethylene is transparent to radiant energy, it might be of value to incorporate polymers that are less transparent at infrared wavelengths.

## 1. Introduction

Low birth weight (LBW) is defined by the World Health Organization as a birth weight of a liveborn infant of 2,499 g or less. Hypothermia increases morbidity and mortality in low-birth-weight (LBW) neonates. A survey [1] of a large, multicenter cohort of LBW neonates found that for each 1°C decrease in body temperature, the likelihood of late-onset sepsis increased by 11% and the likelihood of death increased by 28%. One segment of particular concern is the head, which may account for up to 30% of the total body heat loss (BHL) [2].

According to the American Academy of Pediatrics and American Heart Association's guidelines [3], hypothermia in very LBW neonates (i.e., those weighing less than 1500 g) can be prevented by wrapping with a plastic bag. However, several studies [1, 4–6] have shown that these infants' body temperature increases on admission to the neonatal intensive care unit, increasing the incidence of overheating [6–9]. The cause of this potential adverse event is subject to debate but may reflect the maternal body temperature, infection [7], or interunit differences in use of the plastic bag [8, 10] rather than a direct effect of occlusive wrapping per se. Several researchers [1, 4, 11, 12] have called for further investigation of the efficacy of this approach.

Occlusive bags are used less frequently during surgery outside the incubator [13, 14], even though these procedures are associated with a greater risk of body cooling. In particular, the head (which obviously is not covered by the bag) is exposed to cooler room air. Recommended ways of preventing a drop in body temperature include increasing the room air temperature to 29°C (with a relative air humidity (RH) of 40–60%) and using a radiant heater [15]. However, these procedures increase the risk of dehydration [16]. Wrapping the neonate reduces evaporative heat losses but increases regional skin temperatures and thus radiant heat losses, since polyethylene barely absorbs any infrared radiation. Knowledge of the magnitude of heat exchanges affecting covered and noncovered regions would be of value in assessing the benefits of occlusive plastic bag use during the placement of a percutaneous catheter, a procedure that must always be performed in a timely manner. This can be only achieved by applying a mathematical model based on basic physical principles and verified heat transfer coefficients for the individual body segments. The absence of detailed literature data on these exchanges prompted us to carry out the present study of polyethylene bag use in premature neonates.

## 2. Methods

### 2.1. Clinical Study

Preterm newborn infants born before 32 weeks of gestation were included before the 12th hour of life, after we received written informed consent from their parents. None had congenital anomalies, special diets, or neurological, gastrointestinal, cardiovascular, or respiratory disorders. The protocol was approved by the local institutional review board (*Comité de Protection des Personnes Nord-Ouest II*, Amiens, France; reference: 2010-A00337-32). Thirty premature but clinically stable neonates were included. They were cared for, that is, nursed at thermoneutrality [17] in a closed incubator (Caleo®, Dräger, Lübeck, Germany). The target abdominal skin temperature (*T*_ab_) was set to 36.8°C, with an RH of 70% and an air velocity <0.10 m·s^−1^. Prior to placement of a percutaneous vena cava catheter, the front of the incubator was opened, and the mattress support tray was pulled forward so that the infant was situated outside the incubator. The infant lay in the supine position with the face straight up. An enclosing canopy was formed over the aperture by attaching a surgical sheet to the top of the incubator and the outer edge of the mattress. The air surrounding the neonate therefore mixed to some extent with the cooler room air, which was maintained at 24°C (RH: 40–50%). Although the surgical procedure lasted between 75 and 90 min, only data for the first 60 minutes were recorded and analyzed.

A “wrapped” thermal management protocol was implemented for 14 neonates (the W group: mean ± standard deviation (SD); body mass: 1052 ± 26 g; mean gestational age: 29 ± 0.8 weeks; mean postnatal age: 4.7 ± 0.4 days). The infants were wrapped in a 20 × 30 cm impermeable, sterile polyethylene plastic bag (GE Medical Systems; thickness: 50 *μ*m, mass: 5 g; infrared transmittance: 98%) during the catheter placement. The top of the bag was closed over the shoulders, leaving the head and the neck exposed. A small hole was cut in the bag to enable vascular access to the right arm. The W group was compared with a control group of 16 “nonwrapped” neonates (the NW group; mean body mass: 1065 ± 24 g; mean gestational age: 30 ± 0.7 weeks; mean postnatal age: 4.4 ± 0.3 days). All the neonates wore a diaper covering about 15% of the total body skin surface area.

The air temperatures (*T*_*a*_) inside and outside the incubator were continuously measured with thermistors (series 40 BA, Yellow Springs Instruments; accuracy: ±0.10°C). The RH was measured with a hygrometer (RHU 207®, General Eastern, Mulhouse, France; error range < 5% over the RH operating range of 20–90%), as recommended [3]. Air velocity was recorded with a hot-wire anemometer (TESTO 490®, Forbach, France; accuracy: ±0.05 m·s^−1^).

Skin temperatures (*T*_sk_) were measured by infrared thermometry using a camera (Thermovision 550®, AGEMA, Danderyl, Sweden; sensitivity: ±0.10°C at 30°C; accuracy: ±2°C between 20 and 250°C). Before each measurement, the camera's emissivity was calibrated against *T*_ab_. Infrared scans were acquired every 30 s and digitized with a microcomputer (Therma CAM™ reporter, FLIR Systems, Boston, MA, USA). Two investigators separately evaluated the temperature data. Infrared thermometry avoids the stress caused by attachment of skin probes and minimizes interference with care procedures. To avoid undue disturbances, all regional *T*_sk_ values were recorded during the last 5 minutes of the baseline period in the incubator and at the end of the catheter placement procedure.

*T*_ab_ was continuously measured with a temperature probe placed on the neonate's right lateral abdomen and covered with reflective tape (as used in routine clinical practice). This standard measurement site provides a continuous indication of the deep tissue temperature (due to the near zero heat flux) and is known to be sufficient for monitoring a neonate's thermal status during care [18, 19].

### 2.2. Skin Heat Losses (SHLs): The Mathematical Model

SHLs occur through convection (*C*), radiation (*R*), evaporation (*E*), and conduction (*K*). Since these heat losses are dependent on each body segment, they are calculated separately (in kJ·h^−1^·kg^−1^) for the head, the trunk, the lower limbs, and the upper limbs during the baseline period and again at the end of the catheter placement. The neonate's skin surface was thus divided into a head region (comprising 28% of the total body skin surface area), a torso region (23%), the upper limbs (19%), and the lower limbs (30%):

(1) $$\begin{array}{c} C=h_{c}\left(\;T_{a}-{\bar{T}}_{sk}\;\right)F_{cl}A_{D}Wt^{-1}, \end{array}$$

where *h*_*c*_ is the convective heat transfer coefficient (in kJ·h^−1^·m^−2^·°C^−1^), $T_{a}-{\bar{T}}_{sk}$ is the difference between the air temperature and mean skin temperature (in °C), *F*_cl_ is the dimensionless reduction factor due to clothing thermal insulation, *A*_*D*_ is the neonate's skin surface area (in m^2^), and Wt is the body mass (in kg):

(2) $$\begin{array}{c} R=\sigma \varepsilon_{sk}A_{r}\left(\;{\bar{T}}_{r}-{\bar{T}}_{sk}\;\right)F_{cl}A_{D}{Wt}^{-1}, \end{array}$$

where ${\bar{T}}_{r}-{\bar{T}}_{sk}$ is the difference between the mean radiant temperature and the mean skin temperature of the body segment (in °C), *σ* is the Stephan-Boltzmann constant (20.45·10^−8^ kJ·h^−1^·m^−2^·°C^−1^), *ε*_sk_ is the skin emissivity (typical value: 0.97), and *A*_*r*_ is the effective radiant surface area (in %); ${\bar{T}}_{r}$ has been assessed by Décima et al. [20]:

(3) $$\begin{array}{c} E=h_{e}\omega \left(\;P_{SH2O}-P_{aH2O}\times RH\;\right)F_{pcl}A_{D}Wt^{-1}, \end{array}$$

where *h*_*e*_ is the evaporative heat transfer coefficient = 16.7*h*_*c*_ (in kJ·h^−1^·m^−2^·mb^−1^), *ω* is the skin wettedness (0.20 when no sweating occurs), *P*_SH2O_ − *P*_aH2O_ is the difference (in mb) between the saturated vapor pressure of water at skin temperature (*P*_SH2O_) and at air temperature (*P*_aH2O_), and *F*_pcl_ is the dimensionless reduction factor for water vapor transfer due to clothing insulation.

In a previous study, conduction was found to correspond to 6.45% of the radiant, convective, and evaporative heat loss [21].

The equations defining *R*, *C*, *E*, and *K* were applied once for each body segment. The parameters in the equations (Table 1) have been successfully assessed with an anthropomorphic, sweating thermal mannequin with the same geometrical shape as the population of neonates included in the present study [22, 23]. Given the lack of literature data, *F*_cl_ and *F*_pcl_ were assessed in a separate session (as recommended by the ISO 9920 standard, albeit in adults [24]).

The total BHL is the weighted sum of the SHLs and the evaporative (*E*_resp_) and convective (*C*_resp_) respiratory heat losses (as assessed by Museux et al.) [25]. The weighted factors take into account the surface area of each body part, relative to the total body surface area:

(4) $$\begin{array}{c} BHL=\left(\;\pm R\pm C\pm K+E\pm C_{res}+E_{res}\;\right). \end{array}$$

At the thermoneutral temperature in the incubator, the body reaches thermal equilibrium with the environment; metabolic heat production (*M*) is at its lowest and is equal to the total BHL.

### 2.3. Statistical Analysis

Differences between W and NW groups were assessed in Student's *t*-test at baseline and at the end of the catheter placement procedure. The threshold for statistical significance was set to *p* < 0.05. *t* values quoted with their corresponding degrees of freedom as subscripts. Quantitative variables are reported as the mean ± SD. To describe the thermal instability during the surgical procedure, the coefficients of variation (CV) for the temperatures were calculated from the ratio between the SD and the mean.

## 3. Results

During catheter placement, the neonates experienced a rapid decrease in *T*_*a*_ within the enclosing canopy (from 35.0°C to 29.9°C, over 5 min). This was due to mixing between incubator air and room air and was associated with a fall in ${\bar{T}}_{r}$ from 31.2°C to 27.6°C. The RH was 45%, and the air velocity remained below 0.07 m·s^−1^ (i.e., free convection conditions). These ambient parameters did not differ significantly when comparing the two groups.

**Figure 1:**
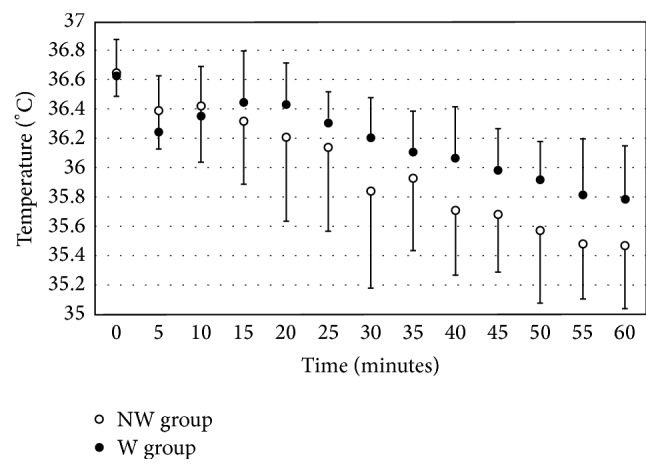
Abdominal skin temperature. Mean ± SD changes over time in abdominal skin temperatures for neonates in the W group (•) and the NW group (∘). ^*∗*^*p* ≤ 0.05.

Figure 1 shows that, in the incubator (i.e., before catheter placement), *T*_ab_ was very similar in the two groups (36.63 ± 0.25°C in the W group versus 36.65 ± 0.16°C in the NW group). During the catheter placement, *T*_ab_ was higher in the W group; after 60 min, this difference was small (0.32°C) but statistically significant (35.79 ± 0.36°C in the W group versus 35.47 ± 0.43°C in the NW group; *t*_28_ = 2.191; *p* = 0.05).

Before catheter placement, the regional *T*_sk_ values did not differ when comparing the two groups (Table 2). At the end of catheter placement, *T*_sk_ values were significantly lower in the W group. In particular, the head *T*_sk_ decreased by 0.39°C in the W group and by 2.64°C in the NW group. The greatest CVs for *T*_sk_ were observed in the NW group (2.4% ≤ CV ≤ 3.8% in the W group versus 4.6% ≤ CV ≤ 6.2% in the NW group, depending on the body site considered).

The relative weights of the various BHL routes did not differ greatly when comparing the values inside the incubator (Table 3). *M* was similar in the two groups (8.65 ± 3.21 kJ·h^−1^kg^−1^ in the W group versus 8.25 ± 3.97 in the NW group). *R* was particularly relevant and accounted for 52-53% of the total BHL. At the end of the catheter placement procedure, outside the incubator, *R* was significantly greater (*t*_28_ = 4.097; *p* ≤ 0.0001) in the W group (difference: 3.44 kJ·h^−1^kg^−1^) than in the NW group. This heat loss was partly balanced by a gain in *E* (2.49 kJ·h^−1^·kg^−1^  *t*_28_ = 8.991; *p* ≤ 0.0001). The total BHL did not differ between the two groups (15.80 ± 3.57 in the W group versus 14.97 ± 5.53 kJ·h^−1^·kg^−1^ in the NW group).

Compared with the NW group, *R*, *C*, *E*, and *K* increased (*t*_28_ always ≥2.314; 0.01 ≤ *p* ≤ 0.0001) for the head's skin surface area of the head in the W group (Table 4). With the exception of the upper limbs, *R* increased for all the covered body segments (*t*_28_ was always ≥2.436; *p* ≤ 0.02).

## 4. Discussion

Our present results highlighted a paradox: wrapping the neonate in a polyethylene bag only provides a small thermal benefit, since increases in some regional skin temperatures promote radiant, conductive, and convective heat losses and thus counterbalance the decrease of evaporative heat losses due to the plastic bag. The ability to dissipate heat from the head is particularly relevant for the prevention of thermal stress during catheter placement.

Although catheter placement is an integral part of routine care for LBW neonates in the first days of life, this procedure has received little attention. To the best of our knowledge, the present study is the first to have used a mathematical model to describe the four BHL routes (radiation, conduction, convection, and evaporation) for each of six body segments.

Our model provides an accurate description of the neonate's thermal status. All the parameters included in the model were derived from reliable experimental data from a multisegment thermal mannequin with the same anthropomorphic dimensions as the neonates studied here. This approach reduces uncertainties, so that the parameters governing heat exchanges are as precise as possible. At present, the use of a heated, anthropomorphic mannequin (taking into account the neonate's shape and regional skin thermal heterogeneity) is the most relevant method for determining the values of heat transfer coefficients, mean radiant temperatures, and clothing thermal insulation, especially in unstable thermal environments [22, 23]. The model has already been validated in neonates and provides values similar to those measured from O_2_ consumption at thermoneutrality (the gold standard) [21, 25].

The present study had some limitations. It featured a new, exploratory design applied to neonates during standard clinical management protocol; hence, it was not possible to continuously record regional skin temperatures without disturbing the catheter placement or rectal temperature (which remains the goal standard for assessing an infant's thermal status). However, the zero-heat-flux method used to record *T*_ab_ in the present study is known to be well correlated with the rectal temperature in both stable and unstable thermal environments [18, 19, 26]. This noninvasive measurement site is commonly used, since it is comfortable for LBW neonates and avoids the potential complications associated with a continuous rectal probe (e.g., the risk of rectal perforation) [27].

## 5. Conclusion

Although only a small thermal benefit (when considering *T*_ab_ and whole-body heat loss) was associated with use of an occlusive polyethylene bag, it would be unwise to conclude that this method is not effective for improving the thermal management of neonates during surgery. The bag rapidly impedes evaporative skin cooling and reduces air turbulence around the enclosed skin surface area; this contributes to the neonates' thermal stability as shown by the lower thermal variability of *T*_sk_ in the W group. In uncovered neonates, peripheral vasoconstriction ascribed by the falls in regional *T*_sk_ can also place a functional load on the cardiovascular system.

Our results show that use of a polyethylene bag is associated with increased radiant heat loss not only from the head but also for the rest of the body, since the bag's infrared transmittance is high. This explains why additional radiant heat source is sometimes required to warm the wrapped neonate sufficiently at birth. Our present results suggest that, during a surgical procedure, it would be helpful to use a bag made from polymers with less infrared transmittance (such as polyvinylidene chloride and related products) and that would trap part of the radiant heat emitted by the body. The use of our mathematical model to take account of the different body elements might provide information on the contribution of thermal stress induced by infrared-transparent blankets that only cover a proportion of the body's surface area.

## Figures and Tables

**Table 1:** Parameters used in the mathematical models' equations to calculate regional skin heat exchanges. *h*_*c*_ is the convective heat transfer coefficient kJ·h^−1^·m^−2^·mb^−1^ and *F*_cl_ and *F*_pcl_ are reduction factors due to the thermal insulation provided by clothing (a diaper for the NW group and a diaper and a polyethylene bag for the W group). For the W group, *F*_cl_ was modified according to the transmittance of the polyethylene bag when calculating *R*.

	*h*_*c*_	*A*_Dseg_ (%)	*A*_*r*_ (%)	The NW group		The W group		
				*F*_cl_	*F*_pcl_	*F*_cl_		*F*_pcl_
						Convection	Radiation	Evaporation
Head	8.0	28	71.9	1	1	1	1	1
Trunk	12.3	23	69.4	0.68	0.62	0.63	0.62	0
Upper limbs	15.1	19	86.4	1	1	0.92	0.90	0
Lower limbs	14.6	30	86.5	0.91	0.91	0.91	0.89	0
Whole body	14.6	100	78.3					

The NW group: control, nonwrapped neonates. The W group: wrapped neonates.

**Table 2:** Regional skin temperatures (°C) measured in the incubator (shown in the left part of the table) and at the end of the catheter placement procedure outside the incubator (shown in the right part) for the W and the NW groups. The quoted values are the mean ± SD.

	Before catheter placement		During catheter placement	
	(in the incubator)		(outside the incubator)	
	NW group (°C)	W group (°C)	NW group (°C)	W group (°C)
Head	35.46 ± 1.38	35.55 ± 1.51	32.82 ± 1.84	35.16 ± 0.86
Trunk	34.93 ± 1.14	34.70 ± 1.10	32.33 ± 1.30	34.41 ± 1.17
Upper limbs	32.36 ± 1.53	32.62 ± 1.12	29.95 ± 1.85	31.87 ± 1.21
Lower limbs	34.37 ± 1.05	34.23 ± 1.06	31.57 ± 1.50	34.26 ± 0.68

The NW group: control, nonwrapped neonates. The W group: wrapped neonates.

**Table 3:** Skin heat losses (kJ·h^−1^·kg^−1^) for the NW and W groups (as calculated by the mathematical model) in the incubator and at the end of the catheter placement procedure. *C*_res_ + *E*_res_ = convective + evaporative heat losses from the respiratory tract; BHL: whole-body heat loss; *M*: basal metabolic heat production.

	Before catheter placement		During catheter placement	
	(in the incubator)		(outside the incubator)	
	NW group	W group	NW group	W group
Radiation	−4.31 ± 1.65	−4.58 ± 1.80	−5.75 ± 1.75	−9.19 ± 2.80
Convection	+0.95 ± 0.30	+0.85 ± 0.35	−2.60 ± 1.69	−2.43 ± 1.10
Evaporation	−3.23 ± 1.50	−3.22 ± 0.86	−4.25 ± 0.99	−1.76 ± 0.32
Conduction	−0.43 ± 0.30	−0.41 ± 0.39	−0.81 ± 0.29	−0.86 ± 0.22
*C*_res_ + *E*_res_	−1.23 ± 0.40	−1.29 ± 0.18	−1.56 ± 0.81	−1.56 ± 0.33
BHL = *M*	−8.25 ± 3.97	−8.65 ± 3.21	−14.97 ± 5.53	−15.80 ± 3.57

The NW group: control, nonwrapped neonates. The W group: wrapped neonates.

**Table 4:** Calculated regional body heat losses (kJ·h^−1^·kg^−1^) at the end of the catheter placement.

	Radiation		Convection		Evaporation		Conduction	
	NW group	W group	NW group	W group	NW group	W group	NW group	W group
Head	7.44^*∗*^ ± 3.77	11.4^*∗*^ ± 5.56	4.52^*∗∗∗*^ ± 2.87	8.69^*∗∗∗*^ ± 3.09	4.83^*∗*^ ± 1.44	6.28^*∗*^ ± 1.45	1.08^*∗∗*^ ± 0.50	1.70^*∗∗*^ ± 0.65
Trunk	4.45^*∗*^ ± 0.72	6.18^*∗*^ ± 2.74	1.99 ± 1.08	0 ± 0	6.23^*∗∗∗*^ ± 0.25	0^*∗∗∗*^ ± 0	0.82 ± 0.11	0.52 ± 0.08
Upper limbs	4.07 ± 2.19	7.02 ± 6.29	0 ± 0	0 ± 0	3.51^*∗∗∗*^ ± 0.90	0^*∗∗∗*^ ± 0	0.49 ± 0.36	0.60 ± 0.15
Lower limbs	6.23^*∗∗∗*^ ± 1.04	10.81^*∗∗∗*^ ± 0.76	2.93 ± 2.01	0 ± 0	3.70^*∗∗∗*^ ± 0.56	0^*∗∗∗*^ ± 0	0.83 ± 0.25	0.84 ± 0.05

The NW group: control, nonwrapped neonates. The W group: wrapped neonates. ^*∗*^*P* < 0.05, ^*∗∗*^*P* < 0.01, and ^*∗∗∗*^*P* < 0.001.
